# Supplementary material for: The Probiotic Strain Clostridium butyricum TO-A Produces Butyrate by Utilizing Lactate and Acetate
Source: Int J Mol Sci. 2025 Mar 24;26(7):2951. doi: 10.3390/ijms26072951 (PMC11988312; doi:10.3390/ijms26072951)
Supplement: Supplementary file 1 [file ijms-26-02951-s001.zip › ijms-3525250-supplementary.pdf]

Supplemental methods.

Effect of single addition of lactate or acetate on lactate utilization by CBTOA

To evaluate the effect of single addition of lactate or acetate on lactate utilization by CBTOA, cells were cultured under anaerobic conditions at 37 °C and 150 rpm in PY+A medium, which is PY medium supplemented with 0.5% sodium acetate $3H_2O$, or in PY+D medium, which is PY medium supplemented with 0.5% D-lactic acid.

Table S1. CBTOA-related parameters and organic acid production in PY+A medium and PY+D medium after 16 h of cultivation

(A) CBTOA-related parameters in PY+A medium and PY+D medium after 16 h of cultivation. (B) Concentration and net production of organic acids in PY+A medium and PY+D medium at the start of cultivation (0 h) and after 16 h of cultivation (16 h). n=3. Data are presented as mean ± SD.

A

**Medium**	**pH**	**OD_{600}**	**Total Viable Count (CFU mL^{-1})**	**Spore Count (CFU mL^{-1})**	**Sporulation Rate (%)**
PY+A	6.09 ± 0.08	0.48 ± 0.03	5.80×10^{6}	4.00×10^{4}	0.8
PY+D	5.93 ± 0.04	0.53 ± 0.01	1.48×10^{6}	3.33×10^{4}	2.3

B

**Medium**	**Organic Acid**	**Concentration (mM)**		***p*-Value**	**Net Production (mM)**
		**0 h**	**16 h**		
PY+A	Lactate	3.00 ± 0.10	3.67 ± 0.10	0.001	0.67 ± 0.03
	Acetate	39.38 ± 0.73	40.90 ± 0.89	0.004	1.52 ± 0.16
	Butyrate	0.10 ± 0.05	2.89 ± 0.05	0.0000	2.79 ± 0.02
PY+D	Lactate	43.92 ± 1.89	44.31 ± 2.37	0.601	0.39 ± 1.11
	Acetate	0.87 ± 0.01	2.14 ± 0.05	0.001	1.27 ± 0.06
	Butyrate	0.09 ± 0.01	3.00 ± 0.23	0.002	2.91 ± 0.23

Measurement of D-lactate and L-lactate concentrations in medium without CBTOA inoculation

To evaluate the change in D-lactate or L-lactate concentration in medium without CBTOA inoculation, PY+AD, PY+AL, and PY+ADL medium was cultivated without CBTOA inoculation. Cultivation was carried out under the conditions described in the Growth conditions for CBTOA section above, except for the absence of CBTOA.

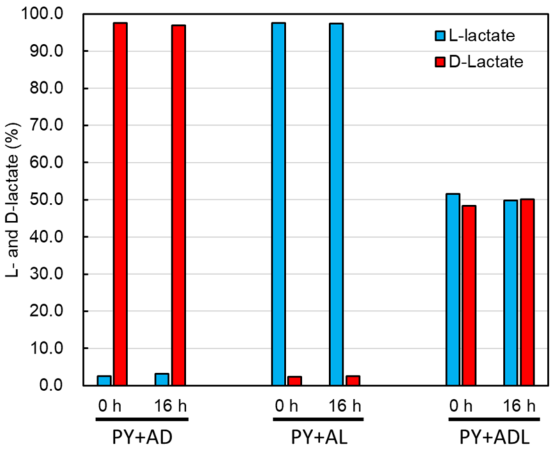

Figure S1. Ratios of lactate enantiomers in various media at the start (0 h) and after 16 h (16 h) of cultivation without CBTOA. The percentages of each lactate enantiomer in the culture medium are shown. Blue bars, L-lactate; red bars, D-lactate.

Table S2. Information on lactate utilization-related genes from various bacteria used in this study

Putative lactate utilization proteins	Bacteria				
	Clostridium butyricum TO-A	*Clostridium butyricum* KNU-L09	*Acetobacterium woodii* DSM 1030	*Anaerostipes hadrus* SSC/2	*Anaerobutyricum hallii*
Lactate racemase	Accession No. ANF13849.1	Accession No. ALP90232.1	Accession No. AFA47666.1		Accession No. SOB71715.1
Electron transfer flavoprotein beta-subunit EtfB	Accession No. ANF13864.1	Accession No. ALP90247.1	Accession No. AFA47662.1	Accession No. WP_008394109.1	Accession No. SOB71712.1
Electron transfer flavoprotein alpha-subunit EtfA	Accession No. ANF13865.1	Accession No. ALP90248.1	Accession No. AFA47663.1	Accession No. WP_008394110.1	Accession No. SOB71713.1
L-lactate permease	Accession No. ANF13866.1	Accession No. ALP90249.1	Accession No. AFA47665.1	Accession No. WP_008394106.1	Accession No. SOB71710.1
D-LDH	Accession No. ANF13867.1	Accession No. ALP90250.1	Accession No. AFA47664.1	Accession No. WP_015530696.1	Accession No. SOB71711.1
Acyl-CoA dehydrogenase	Accession No. ANF13868.1	Accession No. ALP90251.1		Accession No. WP_008394108.1	Accession No. SOB71714.1
